# Incidence and predictors of early and late sudden cardiac death in hospitalized Japanese patients with new‐onset systolic heart failure

**DOI:** 10.1002/joa3.12618

**Published:** 2021-08-18

**Authors:** Yoshiaki Minami, Noriko Kikuchi, Tsuyoshi Shiga, Atsushi Suzuki, Morio Shoda, Nobuhisa Hagiwara

**Affiliations:** ^1^ Department of Cardiology Tokyo Women’s Medical University Tokyo Japan; ^2^ Department of Clinical Pharmacology and Therapeutics The Jikei University School of Medicine Tokyo Japan; ^3^ Clinical Research Division for Heart Rhythm Management Tokyo Women’s Medical University Tokyo Japan

**Keywords:** arrhythmia, heart failure, implantable cardioverter defibrillator, left ventricular ejection fraction, sudden cardiac death

## Abstract

**Background:**

Patients with heart failure (HF) and low left ventricular ejection fraction (LVEF) are at high risk of sudden cardiac death (SCD). Optimal HF treatment can improve LVEF and reduce the risk of SCD. The aim of this study was to evaluate the incidence and predictors of SCD in Japanese patients with new‐onset systolic HF and to investigate factors that affect LVEF improvement.

**Methods:**

We retrospectively studied 174 consecutive hospitalized patients with new‐onset HF and LVEF ≤35% (median age, 66 years; men, 71%). The primary outcome was a composite of SCD, sustained ventricular arrhythmias, and appropriate implantable cardioverter‐defibrillator therapy.

**Results:**

The cumulative rates of meeting of the primary outcome at 3, 12, and 36 months after discharge were 3.9%, 8.1%, and 10.5%, respectively. Atrial fibrillation was a significant predictor of the primary outcome within 12 months after discharge (odds ratio, 5.87; 95% confidence interval [CI], 1.60–21.57). Among 104 patients who completed follow‐up echocardiography within 12 months after discharge, changes in LVEF were inversely associated with SCD (odds ratio/1% increase, 0.78; 95% CI, 0.65–0.93). A QRS duration <130 ms and a B‐type natriuretic peptide level <170 pg/mL were predictors of LVEF improvement to >35% (odds ratio, 3.69; 95% CI, 1.15–11.77; odds ratio, 3.19; 95% CI, 1.33–7.69, respectively).

**Conclusions:**

Our results showed a high incidence of meeting of the primary outcome within 12 months after discharge in hospitalized patients with new‐onset systolic HF. An improved LVEF may reduce the risk of late SCD.

## Graphical abstract

There were high incidences of the primary outcome (sudden cardiac death, sustained ventricular tachycardia/fibrillation, and appropriate implantable cardioverter‐defibrillator therapy) and all‐cause death within 12 months, especially within 3 months, after discharge in hospitalized Japanese patients with new‐onset heart failure and LVEF ≤35%.

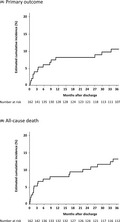

## 1 INTRODUCTION

Sudden cardiac death (SCD) is a major cause of cardiovascular death in patients with systolic heart failure (HF).^1^, ^2^ Low left ventricular ejection fraction (LVEF) is a potential indicator of SCD in patients with myocardial infarction and/or HF.^3^, ^4^, ^5^, ^6^ Previous landmark trials have shown that the use of implantable cardioverter defibrillators (ICDs) reduced all‐cause mortality, including that from SCD, in patients with systolic HF or prior myocardial infarction.^5^, ^6^, ^7^

However, previous clinical trials reported that early implantation of ICDs did not reduce mortality in patients with myocardial infarction.^8^, ^9^ There have been no clinical trials evaluating the benefit of early implantation of ICDs in patients with new‐onset nonischemic HF.^10^ Optimal medical therapy or interventions such as revascularization could improve LVEF within several months and improve prognosis in patients with new‐onset systolic HF or myocardial infarction,^11^, ^12^, ^13^, ^14^ and they would not depend on the presence of an indication for ICD implantation. An observational study reported that LVEF improvement is associated with a decrease in the risks of all‐cause mortality and appropriate shocks in HF patients receiving ICDs for the primary prevention of SCD.^15^

Previous studies revealed that nonischemic etiology, as well as short HF duration/new‐onset HF, are associated with an early improvement in LVEF in patients with systolic HF.^16^, ^17^, ^18^ In Japan, the prevalence of ischemic HF is approximately 30% of hospitalized patients with HF, and nonischemic HF is more common.^19^, ^20^, ^21^ Moreover, most patients with acute myocardial infarction receive early coronary revascularization, and a low prevalence of SCD in Japanese patients with myocardial infarction has been reported.^22^ However, there have been few reports regarding the incidence of SCD during the early period after hospital discharge or on how long we should protect against SCD in Japanese patients with new‐onset HF and low LVEF. The aim of this study was to evaluate the incidence and predictors of early and late occurrences of SCD or sustained ventricular tachycardia (VT)/fibrillation (VF) after discharge in hospitalized Japanese patients with new‐onset systolic HF and to investigate factors that affect LVEF improvement.

## 2 METHODS

### 2.1 Patients

**FIGURE 1:**
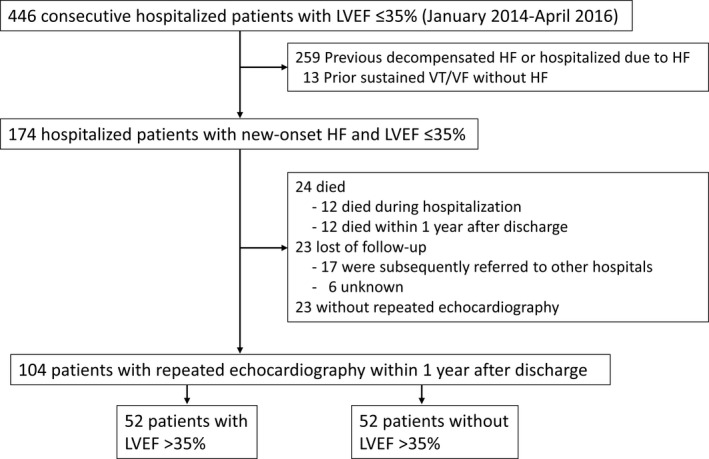
Flow diagram of the study patients. HF, heart failure; LVEF, left ventricular ejection fraction; VT, ventricular tachycardia; VF, ventricular fibrillation

In this retrospective observational study, 174 consecutive hospitalized patients with new‐onset HF and LVEF ≤35% who were admitted to our hospital between January 2014 and April 2016 were enrolled. We searched the inpatient database of our hospital and then confirmed that the patients had an LVEF of ≤35% by echocardiography during hospitalization. Among these patients, we identified consecutive patients with new‐onset (de novo) HF in whom LVEF ≤35% was detected during the index hospitalization by reviewing the patient medical records. New‐onset HF was defined as a first‐time diagnosis of HF. HF was defined as new or worsening signs and symptoms of HF, such as fatigue, dyspnea, edema, elevated venous pressure, crepitations, and additional use of diuretic drugs or treatment with intravenous inotropes and/or vasodilators, noninvasive positive pressure ventilation, respirators, or mechanical support. We excluded patients if the previous hospitalization for worsening HF was documented. Moreover, we also excluded patients who had a history of sustained VT/VF (Figure 1).

We collected information on baseline clinical characteristics and treatment during hospitalization from the medical records of our hospital. Acute myocardial infarction was defined on the basis of the fourth universal definition.^23^ Ischemic cardiomyopathy was defined as LV systolic dysfunction that results from coronary artery disease. Valvular heart disease was defined as a structural abnormality, at least of moderate grade, of cardiac valves or a history of valve surgery. Nonischemic cardiomyopathies were defined as structural and functional abnormalities of the ventricles in the absence of other causes of myocardial dysfunction. Hypertensive heart disease was defined as LV hypertrophy and dysfunction in the absence of other causes such as hypertrophic cardiomyopathy in patients with hypertension.

This study was approved by the institutional review board of Tokyo Women’s Medical University.

### 2.2 Outcomes

The primary outcome was a composite of SCD, sustained VT/VF, and appropriate ICD therapy for VT/VF. SCD was defined as a nontraumatic, unexpected death occurring within 1 h of the onset of symptoms or an unexpected death within 24 hours of having last been seen as well. Secondary outcomes included all‐cause death and the recovery rate of LVEF in 104 patients who completed follow‐up echocardiography within 12 months after discharge. The LV volume and LVEF were measured using the modified Simpson’s method. An improvement in LVEF was defined as more than 35%. Patients were followed until death or June 2020.

Among our patients, six patients received ICDs at hospital discharge, and one patient received an ICD after the index hospitalization. Detection and therapy zones for VF (261–280 ms, 8/12 or 30/40 intervals) and VT (351–353 ms, 28–48 intervals) were programmed. Antitachycardia pacing therapies were activated in six patients. An ICD shock was delivered when triggered by VF or if the antitachycardia pacing failed to terminate VT.

### 2.3 Statistical analysis

Statistical analyses were performed using IBM SPSS Statistics software, version 22.0 (IBM Corp.). Summary values are presented as the number of patients or as the medians with interquartile ranges. The chi‐square test or Fisher’s exact test was used to compare categorical variables. The Kaplan–Meier method was used to estimate the cumulative proportion of the event‐free rate. Multivariate Cox regression analysis estimated the relationships between baseline clinical characteristics and the primary outcome that occurred within 12 months after hospital discharge. Clinical variables were advanced age (>60 years), male sex, ischemic etiology, New York Heart Association Class II, estimated glomerular filtration rate (eGFR) <60 mL/min/1.73 m^2^, nonsustained VT, atrial fibrillation, nonuse of β‐blockers, and nonuse of angiotensin‐converting enzyme inhibitors (ACEIs)/angiotensin receptor blockers (ARBs), which are related to SCD. To evaluate the influence of LVEF improvement on subsequent outcomes, the Cox proportional hazards model adjusting for age, sex, risk factors, renal function, and medications was evaluated in patients with an improved LVEF >35% compared with patients without LVEF improvement. Univariate and multivariate Cox regression analyses estimated the relationships between baseline clinical characteristics and an improved LVEF of >35%. Clinical variables were selected based on previously reported predictors of clinical outcomes, such as age <60 years, female sex, eGFR ≥60 mL/min/1.73 m^2^, plasma brain natriuretic peptide (BNP) level <170 pg/mL,^24^, ^25^ ischemic etiology, use of β‐blockers, use of ACEIs/ARBs, use of cardiac resynchronizing therapy (CRT), and electrocardiographic and echocardiographic parameters. Multivariate analysis was performed using a forward stepwise method, with entry or removal based on a *P* value of <.05. A *P* value of <.05 was considered to be statistically significant.

## 3 RESULTS

### 3.1 Patient characteristics

The median age was 66 years, and the proportion of males was 71%. Among the patients, nonischemic cardiomyopathy was the most common etiology, and ischemic etiology accounted for 28% of cases. Persistent atrial fibrillation was observed in 25% of patients, and nonsustained VT was detected in 34% of patients during hospitalization (Table 1).

**TABLE 1:** Patient characteristics (*n* = 174)

Age (y)	66 (52‐78)
Male	123 (71%)
Body mass index (kg/m^2^)	23.6 (20.8‐26.7)
Underlying heart disease	
Acute myocardial infarction	9 (5%)
Ischemic cardiomyopathy	40 (23%)
Nonischemic cardiomyopathy	87 (50%)
Hypertensive heart disease	24 (14%)
Valvular heart disease	14 (8%)
Family history of sudden death	8 (5%)
NYHA functional class at admission (II/III/IV)	72/53/49
Plasma BNP level at admission (pg/mL)	772 (342‐1385)
Hemoglobin at admission (g/dL)	13.6 (11.6‐15.0)
eGFR (mL/min/1.73 m^2^)	53 (28‐69)
Comorbidities	
Hypertension	104 (60%)
Diabetes mellitus	53 (30%)
Dyslipidemia	81 (47%)
Echocardiography	
LVEDD (mm)	59 (54‐66)
LVESD (mm)	50 (45‐58)
LVEF (%)	30 (25‐33)
Persistent atrial fibrillation	44 (25%)
Nonsustained VT during hospitalization	60 (34%)
Electrocardiographic parameters at admission	
Heart rate	90 (75‐107)
QRS duration (ms)	100 (90‐116)
QTc (ms)	431 (416‐448)

Note

Values are *n* (%) or median (interquartile range).

Abbreviations: BNP, brain natriuretic peptide; eGFR, estimated glomerular filtration rate; LVEDD, left ventricular end‐diastolic dimension; LVEF, left ventricular ejection fraction; LVESD, left ventricular end‐systolic dimension; NYHA, New York Heart Association; QTc, corrected QT interval; VT, ventricular tachycardia.

During hospitalization, 27 (16%) patients underwent percutaneous coronary intervention or cardiac surgery, and 9 (5%) patients received implanted CRT and/or ICD therapy. Regarding medications prescribed during hospitalization, more than 80% of patients started β‐blockers and ACEIs/ARBs (Table 2).

**TABLE 2:** Procedures during hospitalization and medications at discharge

	*n* = 174
Procedures	
PCI	18 (10%)
Cardiac surgery	9 (5%)
Catheter ablation for AF/AFL	7 (4%)
CRT‐P	3 (2%)
CRT‐D/ICD	6 (3%)
PCPS	1 (1%)
LVAS	3 (2%)
Patients who were discharged alive	*n* = 162
Medications at discharge	
β‐blockers	140 (86%)
ACEIs/ARBs	134 (83%)
MRAs	73 (45%)
Loop diuretics	101 (62%)
Digoxin	14 (9%)
Amiodarone	30 (19%)
Statins	57 (35%)

Note

Values are *n* (%).

Abbreviations: ACEIs, angiotensin converting enzyme inhibitors; AF, atrial fibrillation; AFL, atrial flutter; ARBs, angiotensin receptor blockers; CRT‐D, cardiac resynchronization therapy with a defibrillator; CRT‐P, cardiac resynchronization therapy with biventricular pacing; ICD, implantable cardioverter defibrillator; LVAS, left ventricular assist system; MRAs, mineralocorticoid receptor antagonists; PCI, percutaneous coronary intervention; PCPS, percutaneous cardiopulmonary support.

### 3.2 Outcomes

**FIGURE 2:**
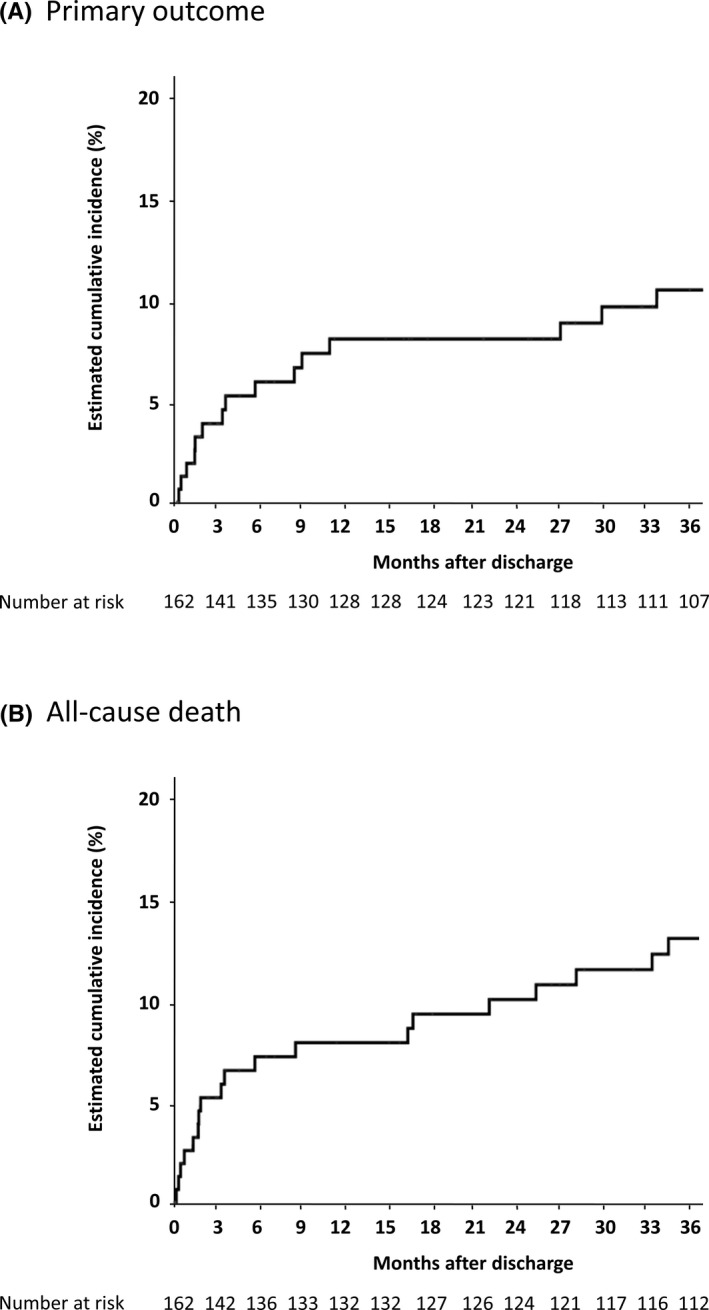
Kaplan–Meier curves of the primary outcome (a composite of sudden cardiac death, sustained ventricular tachycardia/fibrillation, and appropriate implantable cardioverter‐defibrillator therapy) (A) and all‐cause death (B) in patients with new‐onset systolic heart failure who were discharged alive

During the median follow‐up period of 51 [14–64] months, 11 (6%) patients experienced SCD, 4 (2%) patients survived sustained VT/VF, and 3 (2%) patients received appropriate ICD therapy. Among them, one patient experienced sustained VT, received a CRT plus ICD (CRT‐D), and subsequently experienced an appropriate shock after discharge. A total of 43 patients died including 12 in‐hospital deaths. Kaplan–Meier curves for the primary outcome and all‐cause death are shown in Figure 2. The cumulative rates of the meeting of the primary outcome at 3, 12, and 36 months were 3.9%, 8.1%, and 10.5%, respectively, and the cumulative all‐cause death rates at 3, 12, and 36 months were 5.2%, 7.9%, and 13.0%, respectively.

The cause of death and occurrence of VT/VF according to in‐hospital stay and time after hospital discharge are shown in Table S1. Within 3 months after discharge, 6 (3.7%) of 162 patients who were discharged alive experienced SCD or received appropriate ICD therapy. A total of 11 patients (6.8%) experienced SCD, had sustained VT/VF, or received appropriate ICD therapy within 12 months after discharge, and the details of these patients are presented in Table S2. The etiologies of heart disease vary, and 5 patients experienced NSVT during hospitalization. Basal rhythm in 6 of 11 patients was atrial fibrillation at discharge. Although 9 of 11 patients received β‐blockers, only 2 patients took the guideline‐recommended maximum dose of β‐blockers and the others took <50% of the maximum dose. Multivariate analysis showed that atrial fibrillation was a significant factor associated with the occurrences of SCD and ventricular arrhythmias within 12 months after discharge (Table 3).

**TABLE 3:** Multivariate predictors of sudden cardiac death and sustained ventricular arrhythmias that occurred within 12 mo after hospital discharge

Variable	OR	95% CI	*P* value
Age >60 y	1.00	0.96‐1.05	.82
Male gender	0.48	0.13‐1.70	.25
Ischemic etiology	2.07	0.41‐10.55	.81
NYHA class II	1.45	0.35‐6.01	.61
eGFR <60 mL/min/1.73 m^2^ at discharge	0.45	0.12‐1.68	.24
Nonsustained VT	1.17	0.34‐4.07	.80
Atrial fibrillation	5.87	1.60‐21.57	.01
No β‐blockers	1.45	0.25‐8.55	.68
No ACEIs/ARBs	1.75	0.31‐10.00	.53

Abbreviations: ACEIs, angiotensin‐converting enzyme inhibitors; ARBs, angiotensin receptor blockers; CI, confidence interval; CRT, cardiac resynchronization therapy; eGFR, estimated glomerular filtration rate; OR, odds ratio; NYHA, New York Heart Association; VT, ventricular tachycardia.

### 3.3 Follow‐up LVEF

Among patients who remained alive after discharge, 104 patients received follow‐up echocardiography within 12 months after discharge (median of 6 months after discharge). The mean LVEF significantly improved from the index hospitalization to posthospitalization (from 30% [25–33] to 37% [30–44]), and 52 of 104 patients (50%) demonstrated an improvement in LVEF to >35% on follow‐up echocardiography. The SCD trend was a lower incidence in patients with an LVEF improved to >35% than in patients without an LVEF improved to >35% (0/52 vs 4/52, *P* = .06). The odds ratio for SCD was 0.78 (95% CI, 0.65–0.93) for every 1% increase in LVEF. The incidence of all‐cause death was lower in patients with an LVEF improved to >35% than in patients without an LVEF improved to >35% (3/52 vs 11/52, *P* = .02). The odds ratio for all‐cause death was 0.91 (95% CI, 0.84–0.97) for every 1% increase in LVEF. Multivariate analysis showed that a narrow QRS duration (<130 ms) and low BNP level (<170 pg/mL) at discharge could be predictors of an LVEF improvement to >35% (Table 4).

**TABLE 4:** Factors associated with improved left ventricular ejection fraction

Variable	Univariate analysis			Multivariate analysis		
	OR	95% CI	*P* value	OR	95% CI	*P* value
Age <60 y	1.47	0.68‐3.19	.33			
Female gender	0.74	0.31‐1.79	.50			
Ischemic etiology	0.89	0.35‐2.26	.81			
Baseline LVEF >30%	1.72	0.79‐3.73	.17			
Baseline LVEDD <60 mm	1.36	0.63‐2.98	.73			
Baseline QRS duration <130 ms	3.46	1.14‐10.53	.03	3.69	1.15‐11.77	.03
Heart rate <75 bpm at discharge	1.35	0.56‐3.26	.50			
Plasm BNP <170 pg/mL at discharge	2.92	1.26‐6.80	.01	3.19	1.33‐7.69	.01
eGFR ≥60 mL/min/1.73 m^2^ at discharge	0.68	0.31‐1.47	.33			
Use of β‐blockers	2.66	0.49‐14.38	.26			
Use of ACEIs/ARBs	0.26	0.05‐1.30	.26			
Use of CRT	0.65	0.11‐4.08	.65			

Abbreviations: ACEIs, angiotensin‐converting enzyme inhibitors; ARBs, angiotensin receptor blockers; BNP, B‐type natriuretic peptide; CI, confidence interval; CRT, cardiac resynchronization therapy; eGFR, estimated glomerular filtration rate; OR, odds ratio; LVEDD, left ventricular end‐diastolic dimension; LVEF, left ventricular ejection fraction.

## 4 DISCUSSION

Our study revealed the following results: (1) during a median follow‐up of 51 months, 17 (9.8%) patients met the primary outcome (SCD, sustained VT/VF, or appropriate ICD therapy) among 174 patients with new‐onset systolic HF (LVEF ≤35%), nearly half of which occurred within 3 months after discharge; (2) the cumulative rates of meeting of the primary outcome at 3 and 12 months were 3.9% and 8.1%, respectively, among 162 patients who were discharged alive; (3) among 104 patients who had available follow‐up echocardiographic data, 50% showed an improvement in LVEF to >35% within 12 months after discharge; (4) patients with an improved LVEF had significantly lower rates of SCD or all‐cause mortality than patients without an improved LVEF; and (5) a narrow QRS duration and low plasma BNP level were independent predictors of improved LVEF.

### 4.1 Early incidence of ventricular arrhythmia

Recent guidelines recommended that ICDs should be implanted after 3 months or more of optimal medical therapy for patients with HF and LVEF ≤35%.^26^, ^27^ However, the risk of SCD or ventricular arrhythmia does not decrease until LV systolic function improves. During the initiation and optimization of HF therapy, patients with newly diagnosed HF and low LVEF are reported to be at high risk of SCD.^28^ During this period, cardioprotective therapies such as β‐blockers have not reached the maximum effective dose because of titration up to the target dose. The challenge is how to protect patients from SCD and sustained VT/VF during this period. Therefore, a wearable cardioverter defibrillator (WCD) is indicated for a limited period in patients at high risk of SCD.^26^

Several observational studies of patients with WCDs showed that 1.6%–4.8% received an appropriate WCD shock within 2 or 3 months among patients with low LVEF and a high risk of SCD, mostly with newly diagnosed ischemic or nonischemic cardiomyopathies.^29^, ^30^, ^31^, ^32^ In our study, 6 (3.7%) of 162 patients who were discharged alive experienced SCD or received an appropriate ICD shock within 3 months after discharge. This result was compatible with previous studies. Our study showed a high incidence of SCD or sustained VT/VF within 3 months after discharge as well as a relatively high incidence up to 12 months after discharge. In other words, we should treat patients with new‐onset HF and low LVEF, recognizing that they are at high risk of SCD during this period. The mechanisms of early SCD occurrence after hospital discharge might be caused by hemodynamics that are not fully stabilized, electrophysiologic effects due to stretched myocardial fibers, neurohormonal activation such as increased sympathetic activity, rapid atrial fibrillation, inflammation, and transient ischemia or electrolyte imbalance that are not yet well controlled by medical HF treatment. In this study, half of the patients who met the primary outcome within 1 year after discharge had atrial fibrillation. Atrial fibrillation was also a significant factor that affected the early occurrence of SCD and ventricular arrhythmias in this study. Atrial fibrillation is known to be a potential risk factor for SCD in patients with several heart diseases such as HF, hypertrophic cardiomyopathy, and prior myocardial infarction.^33^ Although the mechanism of the relationship between atrial fibrillation and SCD is not fully understood, atrial fibrillation with rapid and irregular heart rate may induce ventricular arrhythmias via electrical and structural remodeling of ventricular myocytes. The effect of β‐blockers on mortality is diminished for patients with systolic HF and atrial fibrillation.^34^, ^35^ It is controversial as to how long after the initiation of HF therapy in patients with new‐onset HF there is a risk of SCD, but from our results, it cannot be concluded that a 3‐mo risk reassessment is sufficient.

### 4.2 Changes in LVEF and outcome

Our study showed that 50% of patients who underwent follow‐up echocardiography showed an improvement in LVEF to >35% within 12 months after discharge. The Intervention in Myocarditis and Acute Cardiomyopathy (IMAC)‐2 trial reported that 65% of patients with newly diagnosed nonischemic cardiomyopathy and LVEF <40% showed an improved LVEF 6 months after initiation of cardioprotective therapy including β‐blockers and ACEIs/ARBs.^11^ Another observational study showed that 43% of patients with new‐onset HF and LVEF <30%, of whom 76% had a nonischemic etiology, showed an improvement in LVEF to >35% after 6 months of cardioprotective therapy.^12^ Our results in patients with new‐onset HF, among whom 72% had a nonischemic etiology, were comparable with the results of these studies. Although nonischemic etiology was not a statistically significant factor for LVEF improvement, half of the patients with nonischemic HF improved LVEF to >35% (41 of 81 patients). In our study, a narrow QRS and low BNP levels at discharge were independent predictors of improved LVEF. A previous study reported that baseline prolonged QRS duration was associated with high morbidity and mortality after discharge in hospitalized patients with HF and low LVEF.^36^ The beneficial outcome observed in patients with a narrow QRS duration may be partially due to an improvement in LVEF. Low BNP levels, which suggest less myocardial damage, are also reported to be a predictor of improvement in LVEF.^18^

In our study, patients with improved LVEF to >35% tended toward a lower risk of SCD, but the trend was not conclusive because the occurrence of SCD was quite low. Several studies have shown that an improved LVEF may contribute to a reduced risk of ventricular arrhythmia and mortality.^37^ Interestingly, no SCD was observed in patients with an improved LVEF. This result may partially explain the negative findings from randomized controlled trials of ICDs in patients with nonischemic HF and low LVEF receiving optimal cardioprotective therapy.^38^, ^39^ Moreover, nonpharmacological management, including cardiac rehabilitation, diet (eg, sodium intake and fluid restriction), nutrition, treatment adherence, and psychological support, may significantly impact patient stability, functional capacity, and quality of life as well as mortality, including that from SCD, for patients with HF and low LVEF. Improvement in LVEF will be a therapeutic target for SCD prevention in patients with systolic HF, and WCDs will be a useful tool for Japanese patients with new‐onset systolic HF who initiate HF therapy.

### 4.3 Study limitations

Our study was a single‐center, retrospective, observational design. To minimize selection bias, we enrolled consecutive patients, but 23 patients were lost to follow‐up. The number of patients was relatively small. Although only 6 patients received CRT‐D/ICD, ICD detection, and therapy programming were not identical. Nonessential ICD therapy for the possibility of self‐terminating/nonsustained VT could not be excluded. Because the incidence of late SCD (later than 12 months after hospital discharge) was low, the effect of an improvement in LVEF to >35% on late SCD or significant clinical factors that predict late SCD could not be demonstrated from this small sample size study. The patients in this study were hospitalized during the period when ivabradine and angiotensin receptor–neprilysin inhibitors were not available in Japan. At present, these drugs may also have a contributing role in improving LVEF.

## 5 CONCLUSIONS

Our results showed a high incidence of meeting of the primary outcome criteria within 12 months, especially within 3 months, after discharge in hospitalized patients with new‐onset systolic HF. An improvement in LVEF to >35% within 12 months after discharge was a significant factor related to reduced risks of subsequent SCD and all‐cause mortality.

## CONFLICT OF INTEREST

MS is an endowed chair of Biotronik, Boston‐Scientific, Medtronic, and St. Jude Medical. The remaining authors have no conflicts of interest to declare.

## ETHICAL APPROVAL

IRB approval number: 2020‐0022, IRB approval date: 2021/1/12.

## Supporting information

Table S1

Table S2
