# Supplementary material for: Incidence and predictors of early and late sudden cardiac death in hospitalized Japanese patients with new‐onset systolic heart failure
Source: J Arrhythm. 2021 Aug 18;37(5):1148–55. doi: 10.1002/joa3.12618 (PMC8485812; doi:10.1002/joa3.12618)
Supplement: Supplementary file 1 — Table S1 [file JOA3-37-1148-s001.pdf]

Table S1. Cause of death and ventricular arrhythmic events according to hospitalization and after-discharge period.

Variable	in-hospital	After discharge ~3 months	3~12 months	12~ months
Death	12	8	4	19
Cause of death				
Cardiovascular cause	6	5	2	9
Sudden cardiac death	0	5	2	4
Heart failure	5	0	0	4
Aortic dissection	1	0	0	1
Noncardiac cause	6	3	2	10
Intracranial bleeding	0	1	0	0
Infection	2	0	0	4
Respiratory failure	2	0	0	0
Malignancy	1	2	1	2
Other noncardiac cause	1	0	1	1
Unknown/undetermined cause	0	0	0	3
Sustained VT/VF	3	0	1	0
Appropriate ICD therapy	0	1	2	0

ICD, implantable cardioverter defibrillator; VT, ventricular tachycardia; VF, ventricular fibrillation.
