# Supplementary material for: Incidence and predictors of early and late sudden cardiac death in hospitalized Japanese patients with new‐onset systolic heart failure
Source: J Arrhythm. 2021 Aug 18;37(5):1148–55. doi: 10.1002/joa3.12618 (PMC8485812; doi:10.1002/joa3.12618)
Supplement: Supplementary file 2 — Table S2 [file JOA3-37-1148-s002.pdf]

Table S2. Clinical characteristics of 11 patients who experienced SCD, sus VT or VF within 12 months after hospital discharge.

Age	Sex	Underlying heart disease	LVEF (%)	NYHA class at discharge	eGFR at discharge (mL/min)	BNP at discharge (pg/mL)	Basal rhythm	QRS duration (msec)	QTc (msec)	NSVT during hospital-ization	β-blockers	ACEIs /ARBs	MRAs	Amio-darone	Devices	Event	Days after discharge
66	Male	HHD	20	2	65	1086	AF	110	416	—	+	—	+	+	—	SCD	3
90	Female	DCM	35	3	74	256	AF	92	437	—	+	—	—	—	—	SCD	8
66	Female	OMI	33	2	96	163	SR	84	425	—	—	+	—	—	—	SCD	20
45	Male	Cardiac sarcoidosis	25	2	67	298	SR	120	485	+	+	+	+	—	CRT-D	sus VT	38
89	Female	AMI	33	2	48	526	SR	98	437	—	+	+	+	—	—	SCD	39
72	Female	Post AVR	34	2	38	410	AF	128	430	—	+	+	+	+	—	SCD	55
50	Male	Post AVP	24	2	63	2688	AF	142	452	+	+	+	—	—	—	SCD	106
53	Male	DCM	24	3	64	224	AF	88	407	—	+	+	+	+	—	SCD	171
67	Male	ARVC	24	2	59	1206	SR	100	380	+	—	+	—	+	ICD	sus VT	257
32	Female	Drug-induced CM	35	2	93	367	SR	68	402	+	+	+	+	—	ICD	sus VT	274
85	Male	End-stage HCM	35	2	32	1045	AF	112	463	+	+	+	—	—	—	VF	335

ACEIs, angiotensin-converting enzyme inhibitors; AF, atrial fibrillation; ARBs, angiotensin II receptor blockers; ARVC, arrhythmogenic right ventricular cardiomyopathy; AMI, acute myocardial infarction; AVP, aortic valvuloplasty; AVR, aortic valve replacement; BNP, brain natriuretic peptide; CM, cardiomyopathy; CRT-D, cardiac resynchronization therapy with a defibrillator; DCM, dilated cardiomyopathy; eGFR, estimated glomerular filtration rate; HCM, hypertrophic cardiomyopathy; HHD, hypertensive heart disease; ICD, implantable cardioverter defibrillator; LVEF, left ventricular ejection fraction; MRAs, mineralocorticoid receptor antagonists; NSVT, nonsustained ventricular tachycardia; NYHA, New York Heart Association; OMI, old myocardial infarction; SCD, sudden cardiac death; SR, sinus rhythm; sus VT, sustained ventricular tachycardia; VF, ventricular fibrillation.
